# Efficacy of Musculoskeletal Manipulation for Endoscopic Discomfort in Patients Undergoing Upper Gastrointestinal Endoscopy and Colonoscopy: A Systematic Review and Meta-Analysis

**DOI:** 10.7759/cureus.96660

**Published:** 2025-11-12

**Authors:** Satoru Kitamura, Shinnosuke Komiya, Takao Kaneko, Takahiro Tsuge, Jun Watanabe

**Affiliations:** 1 Department of Internal Medicine, Takemasa-kai Central Hospital, Fukuyama, JPN; 2 Research Support Unit, Scientific Research WorkS Peer Support Group (SRWS-PSG), Osaka, JPN; 3 Holistic Reproductive Antiaging Center, HORAC Grand Front Osaka Clinic, Osaka, JPN; 4 Department of Obstetrics and Gynecology, Kansai Medical University, Osaka, JPN; 5 Department of Rehabilitation, Yamagata Prefectural Central Hospital, Yamagata, JPN; 6 Department of Rehabilitation, Kurashiki Medical Center, Okayama, JPN; 7 Department of Epidemiology, Graduate School of Medicine, Dentistry and Pharmaceutical Sciences, Okayama University, Okayama, JPN; 8 Division of Gastroenterological, General and Transplant Surgery, Jichi Medical University, Shimotsuke, JPN; 9 Center for Community Medicine, Jichi Medical University, Tochigi, JPN; 10 Department of Medicine, Division of Gastroenterology and Farncombe Family Digestive Health Research Institute, McMaster University, Ontario, CAN

**Keywords:** abdominal bloating, abdominal distension, abdominal pain, anxiety, colonoscopy, endoscopy, massage, musculoskeletal intervention, reflexology, upper gastrointestinal endoscopy

## Abstract

Patients undergoing upper gastrointestinal endoscopy (UGIE) and colonoscopy often experience discomfort, including anxiety, abdominal pain, and bloating. The efficacy of musculoskeletal manipulation (e.g., massage) for these symptoms remains unclear. This systematic review and meta-analysis aimed to clarify its efficacy in patients undergoing UGIE and colonoscopy. We searched major electronic databases (MEDLINE (PubMed), CENTRAL, EMBASE, CINAHL, and PEDro) and trial registries up to July 17, 2025, for randomized controlled trials (RCTs). Meta-analyses employed a random-effects model, and risk of bias was formally assessed. Four trials were included. Two trials on reflexology showed a non-significant effect on pre-procedural anxiety, with the evidence being highly variable and leading to very low certainty findings. Two trials on abdominal massage following colonoscopy suggested a possible benefit for reduced abdominal pain, but this finding was limited by substantial heterogeneity. The overall evidence for musculoskeletal manipulation on anxiety, abdominal pain, and bloating is generally low or very low certainty due to imprecision and high risk of bias. While the overall evidence is insufficient for routine clinical recommendations, future high-quality, large-scale trials that standardize outcome assessments are essential to establish definitive conclusions. This review was registered with PROSPERO (CRD420251104522).

## Introduction and background

Upper gastrointestinal endoscopy (UGIE) and colonoscopy are considered gold-standard procedures for diagnosing and essential tools for the endoscopic treatment of gastrointestinal cancers and their precancerous lesions [1,2]. In the United States, approximately 7.5 million upper gastrointestinal endoscopies and 13.8 million colonoscopies are performed annually [3]. While colonoscopy has been shown to reduce colorectal cancer incidence by 31% and colorectal cancer-related mortality by 50%, the uptake rate among invited patients remains only 41% [4]. It is known that patients undergoing colonoscopy and UGIE experience high levels of anxiety [5,6], suggesting that anxiety may be a barrier to screening attendance. Furthermore, physical discomforts such as post-UGIE pharyngeal pain and abdominal pain [7], as well as post-colonoscopy abdominal pain, bloating (subjective sensation of abdominal distension), and general abdominal discomfort [8] also contribute to patient burden, necessitating appropriate interventions.

To alleviate anxiety and pain during endoscopic procedures, various non-pharmacological interventions have been explored. These non-pharmacological approaches may be valuable as alternative or complementary interventions; however, sedation remains the most widely used approach [9]. Previous studies have reported that pre-procedural educational programs can reduce discomfort during UGIE [10], and a combination of anxiety-specific care and seamless care has also shown effectiveness [11]. Music interventions, audiovisual distraction, video information, individualized education, and electroacupuncture have demonstrated statistically significant effects for alleviating anxiety in patients undergoing colonoscopy [12]. Moreover, a previous meta-analysis has indicated that a virtual reality intervention effectively reduced pain and anxiety in patients undergoing either type of endoscopic procedures [13]. While non-pharmacological interventions are explicitly recommended by the British Society of Gastroenterology guidelines [9], many of these interventions are psychological or sensory approaches, and physical non-pharmacological interventions have not been sufficiently investigated to date.

Musculoskeletal manipulation, a physical approach involving manual techniques, such as massage, has recently garnered attention. Musculoskeletal manipulation is defined as a non-pharmacological therapy that "uses hands or devices to apply various manipulations to body tissues, muscles, and bones to promote health, improve circulation, relieve fatigue, and promote healing" by the Medical Subject Headings (MeSH) thesaurus used for PubMed indexing [14]. Indeed, a previous systematic review has reported that reflexology, a type of musculoskeletal manipulation, is effective in reducing anxiety in patients undergoing cardiovascular interventions [15]. To our knowledge, no systematic review of musculoskeletal manipulation specifically targeting patients undergoing gastrointestinal endoscopy has been reported.

Therefore, this systematic review aimed to clarify the extent of research conducted and the efficacy of musculoskeletal manipulation on anxiety, abdominal pain, and abdominal bloating in patients undergoing UGIE and colonoscopy. Furthermore, it sought to evaluate the quality of the existing evidence to guide future research. This simple, non-pharmacological therapy could be applied by various practitioners (such as a healthcare professional, family member, or caregiver) if its clinical implementation is recommended.

## Review

Compliance with reporting guidelines

We conducted this systematic review according to the Preferred Reporting Items for Systematic Reviews and Meta-Analyses 2020 (PRISMA 2020) statement (Appendix A) [16] and a protocol registered with PROSPERO (CRD420251104522). The certainty of evidence was evaluated using the Grading of Recommendations Assessment, Development and Evaluation (GRADE) approach [17].

Eligibility criteria

We included randomized controlled trials (RCTs) that assessed the efficacy of musculoskeletal manipulation on anxiety, abdominal pain, abdominal bloating, and abdominal distension. We did not apply restrictions on language, country, observation period, or publication year. We included published and unpublished papers and conference abstracts. We excluded crossover trials, cluster-randomized trials, and quasi-experimental studies.

Participants were patients aged 18 years or older who were hemodynamically stable and undergoing UGIE or colonoscopy. We excluded patients with emergency endoscopy, drug dependence, or sensory or motor impairment in the manipulation area.

We defined musculoskeletal manipulations as “various manipulations of body tissues, muscles, and bones by hands or equipment to improve health and circulation, relieve fatigue, promote healing" [14]. We searched for the terms related to musculoskeletal manipulation, including bodywork, manual therapy, reflexology, Rolfing, applied kinesiology, chiropractic manipulation, osteopathic manipulation, acupressure, and massage. Intervention site, practitioner type, and intervention duration or timing were not restricted. The control group received routine care, as defined by the original authors.

Outcomes of interest

Primary outcomes were change in anxiety level (measured using scales such as State-Trait Anxiety Inventory: STAI [18] or Depression Anxiety Stress Scales: DASS [19]), abdominal pain (Visual Analogue Scale: VAS or Numeric Rating Scale: NRS), and abdominal bloating (VAS or NRS). Secondary outcomes included all adverse events and changes in abdominal distension measured by objective tools, such as abdominal circumference. For these outcomes, when multiple time points were available, we selected the time point most commonly used across studies.

Search strategies and study selection

We conducted electronic searches on July 16 and 17, 2025, in MEDLINE (via PubMed), the Cochrane Central Register of Controlled Trials (CENTRAL), the Excerpta Medica database (EMBASE), the Cumulative Index to Nursing and Allied Health Literature (CINAHL), and the Physiotherapy Evidence Database (PEDro). We also searched trial registries (the International Clinical Trials Registry Platform and ClinicalTrials.gov) for unpublished trials. The detailed search strategy is provided in Appendix B. We also searched international guidelines [20-22] and the reference lists of eligible studies.

Two independent reviewers (selected from SKi, TK, and TT) screened titles and abstracts, and a full-text eligibility assessment was performed in duplicate after screening. Disagreements were resolved by discussion, with a third reviewer (SKo or JW) acting as an arbiter when necessary.

Data extraction and quality assessment

Two independent reviewers (selected from SKi, TK, and TT) performed data extraction using a pre-tested, standardized form that included information on study design, population, interventions, outcomes, and funding. Two independent reviewers assessed the risk of bias using the Risk of Bias 2.0 tool [23]. Any disagreements were resolved through discussion or by consulting a third reviewer (SKo or JW). We attempted to contact the corresponding authors due to some missing data, including specific information on adverse events. We also attempted to contact the authors of excluded studies, particularly those with unpublished protocols, when contact information was available, but received no response.

Data analysis

We used a random-effects model to pool standardized mean differences for continuous outcomes. We performed meta-analysis using TERA MetaPairwise V1.1.0 [24]. Interventions from multi-arm trials were handled by either combining related interventions or extracting a single intervention for analysis. We estimated the missing mean and the standard deviation using the methods proposed by Wan et al. [25]. We sought to obtain missing data by contacting the authors of all included studies. For studies lacking standard deviations or correlation coefficients, we used a hierarchy of imputation methods [26]. Specifically, we used a correlation coefficient of 0.742, provided by the author of one study [27], to impute data for another study [28]. Missing data for the STAI outcome in study [28] were obtained through direct inquiry to the author. For two studies [29,30], for which the authors did not respond to our inquiries, we assumed a correlation coefficient of r=0.82 based on a previous systematic review [15].

We assessed statistical heterogeneity using the I^2^ statistic (I^2^ values of 0-40%: might not be important; 30-60%: may represent moderate heterogeneity; 50-90%: may represent substantial heterogeneity; 75-100%: considerable heterogeneity [26]) and subsequently determined considerable heterogeneity for the meta-analyzed outcomes (anxiety: I^2^ = 97.1%; abdominal pain: I^2^ = 86.7%). While we planned to explore reasons for substantial heterogeneity (I^2^ > 50%) with subgroup analysis, we did not perform it due to the limited number of studies. Similarly, we did not assess publication bias using a funnel plot or Egger's test due to the limited number of included studies.

Study selection and characteristics

**Figure 1:**
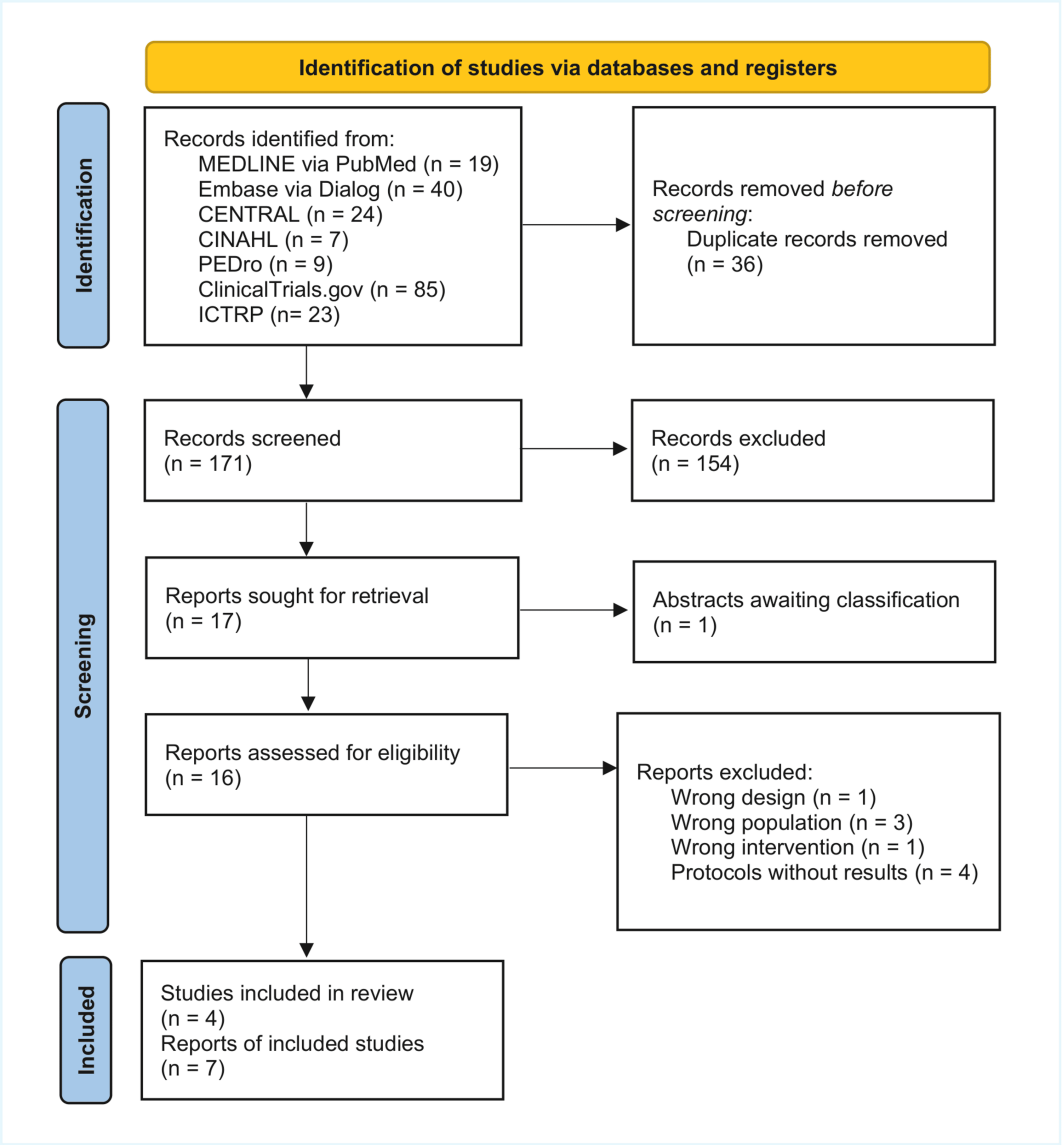
PRISMA 2020 flow diagram for the inclusion of studies in the systematic review Studies included in review (n=4): This is the total number of independent trials or clinical studies included in the meta-analysis. Reports of included studies (n=7): This is the total number of published documents that report on those four included studies. CENTRAL, Cochrane Central Register of Controlled Trials; CINAHL, Cumulative Index to Nursing and Allied Health Literature; EMBASE, Excerpta Medica database; ICTRP, International Clinical Trials Registry Platform; PEDro, Physiotherapy Evidence Database; PRISMA, Preferred Reporting Items for Systematic Reviews and Meta-Analyses

Our electronic searches conducted on July 16 and 17, 2025, initially identified 207 records. After removing 36 duplicates, we screened 171 unique records. Following title and abstract screening, we assessed 16 full-text articles for eligibility. One study was classified as ’awaiting classification’ because the full text was unavailable in Japan. Of the remaining 16 articles, nine were excluded for reasons such as non-relevant study design (n=1), wrong population (n=3), irrelevant intervention (n=1), and protocols without results (n=4) (Appendix C). Ultimately, four trials [27-30] were included in the qualitative synthesis. Studies included in the review (n=4) had published documents (n=7). We identified no additional trials from other sources. The study selection process is detailed in Figure 1.

Outcomes

Table 1 shows the key characteristics of included studies. Two studies [29,30] were conducted in Iran, and two [27,28] were conducted in Turkey. One study [29] received funding from a university.

**Table 1:** Summary of the characteristics of the included studies COI, conflict of interest; DASS, Depression, Anxiety, and Stress Scales; N/A, not available; NRS, Numerical Rating Scale; RCT, randomized controlled trial; STAI, State-Trait Anxiety Inventory; UGIE, upper gastrointestinal endoscopy; VAS, Visual Analogue Scale

Study	Country	Method	Procedure	Follow-up	Study group	Sample size	Age, Mean (SD)	Sex (male: female)	Outcome (measure scale)	Funding/COI
Shaermoghadam et al., 2016 [29]	Iran	Parallel RCT (three-arm)	UGIE	Before procedure	Foot reflexology	30	N/A	N/A	Anxiety (DASS-21)	Zabol University of Medical Sciences
					Hand reflexology	30	N/A	N/A		
					Control	30	N/A	N/A		
Golitaleb et al., 2025 [30]	Iran	Parallel RCT (three-arm)	Colonoscopy	Before procedure	Foot reflexology	33	46.78 (11.89)	19:14	Anxiety (STAI)	No COI
					Hand reflexology	35	42.65 (9.37)	12:23		
					Control	34	44.91 (11.59)	18:16		
Mutlu et al., 2024 [28]	Turkey	Parallel RCT (three-arm)	Colonoscopy	30 minutes after procedure	Abdominal massage	41	49.3 (8.5)	22:19	Abdominal pain (VAS), abdominal distension (abdominal circumference), anxiety (STAI)	No COI
					Control	41	50.6 (9.6)	23:18		
Topcu et al., 2025 [27]	Turkey	Parallel RCT (two-arm)	Colonoscopy	20 minutes after procedure	Abdominal massage	30	58.26 (8.9)	19:11	Abdominal pain (NRS), abdominal bloating (NRS)	No COI
					Control	30	53.26 (15.12)	19:11		

The main findings of this review are summarized in Table 2. Study-specific risks of bias are presented in Table 3 and Appendix D. The overall risk of bias was rated as high for all four included studies.

**Table 2:** Summary of findings; musculoskeletal manipulation compared to control for endoscopic discomfort *The risk in the intervention group (and its 95% confidence interval) is based on the assumed risk in the comparison group and the relative effect of the intervention (and its 95% CI). a Downgraded two levels for very serious risk of bias (e.g., issues beyond just lack of blinding). b No downgrade for inconsistency, as the direction of effect was consistent across all studies despite considerable statistical heterogeneity (I^2^>80%). c Downgraded one level for imprecision. This accounts for the uncertainty stemming from either the wide CI (for meta-analyzed outcomes) or small sample size (for single-study outcomes or adverse effects in synthesized studies). d Downgraded one level for serious risk of bias (e.g., lack of blinding). e Downgraded one level for imprecision due to the lack of a reported confidence interval (for single-study outcomes). GRADE Working Group grades of evidence Low certainty: Our confidence in the effect estimate is limited; the true effect may be substantially different from the estimate of the effect. Very low certainty: We have very little confidence in the effect estimate; the true effect is likely to be substantially different from the estimated effect. CI, confidence interval; DASS, Depression, Anxiety, and Stress Scales; GRADE, Grading of Recommendations Assessment, Development and Evaluation; MD, mean difference; NRS, Numerical Rating Scale; RCT, randomized controlled trial; SD, standard deviation; SMD, standardized mean difference; STAI, State-Trait Anxiety Inventory; UGIE, upper gastrointestinal endoscopy; VAS, Visual Analogue Scale

Outcomes	Anticipated absolute effects* (95% CI)		Relative effect (95% CI)	№ of participants (studies)	Certainty of the evidence (GRADE)	Comments
	Risk with control	Risk with musculoskeletal manipulation				
The effect of musculoskeletal manipulation (reflexology) on pre-procedural anxiety change	-	-	SMD -2.15 SD (4.47 lower to 0.18 higher)	192 (2 RCTs)	⨁◯◯◯ Very low a, b, c	The evidence is very uncertain regarding the effect of reflexology on pre-procedural anxiety change.
The effect of musculoskeletal manipulation (post-colonoscopy abdominal massage) on post-intervention anxiety assessed with STAI Scale from: 20 to 80		Median difference 0.24 lower (-)	Median difference -0.24 (-)	82 (1 RCT)	⨁◯◯◯ Very low c, d, e	Post-colonoscopy abdominal massage may have little to no effect on the effect of abdominal massage on post-intervention anxiety, but the evidence is very uncertain.
The effect of musculoskeletal manipulation (abdominal massage) on post-colonoscopy abdominal pain change	-	-	SMD -1.04 SD (2 lower to 0.05 lower)	142 (2 RCTs)	⨁⨁◯◯ Low b, c, d	The evidence is uncertain regarding the effect of abdominal massage on post-colonoscopy abdominal pain change.
The effect of musculoskeletal manipulation (abdominal massage) on post-colonoscopy abdominal bloating change assessed with NRS Scale from: 0 to 10		MD 0.3 lower (-)	MD -0.3 (-)	60 (1 RCT)	⨁◯◯◯ Very low c, d, e	The evidence is very uncertain regarding the effect of abdominal massage on post-procedural abdominal bloating change.
The effect of musculoskeletal manipulation (abdominal massage) on post-colonoscopy abdominal distension change assessed with: abdominal circumference (cm)		median difference 1 cm lower (-)	median difference -1 cm (-)	82 (1 RCT)	⨁◯◯◯ Very low c, d, e	The evidence is very uncertain regarding the effect of abdominal massage on post-procedural abdominal distension change.
Adverse events of musculoskeletal manipulation (reflexology) - not reported	-	-	-	-	-	Adverse events of reflexology were not mentioned.
Adverse events of musculoskeletal manipulation (abdominal massage)	Low		not estimable	142 (2 RCTs)	⨁⨁◯◯ Low c, d	The evidence is uncertain regarding adverse events of abdominal massage.
	0 per 71	0 per 71				

**Table 3:** Risk of Bias 2.0 (RoB 2.0) assessment of the included randomized controlled trials Domains of RoB 2.0: D1: Bias arising from the randomization process. D2: Bias due to deviations from intended intervention. D3: Bias due to missing outcome data. D4: Bias in measurement of the outcome. D5: Bias in selection of the reported result.

Study	D1	D2	D3	D4	D5	Overall
Shaermoghadam et al., 2016 [29]	Some concerns	High	High	High	High	High
Golitaleb et al., 2025 [30]	Low	Some concerns	Low	High	Low	High
Mutlu et al., 2024 [28]	Some concerns	Low	Low	High	Some concerns	High
Topcu et al., 2025 [27]	Low	Low	Low	High	Low	High

We combined both hand and foot reflexology from two studies [29,30] into a single "reflexology" category. Furthermore, we extracted only the abdominal massage intervention from one study [28] for analysis.

Primary outcomes

**Figure 2:**
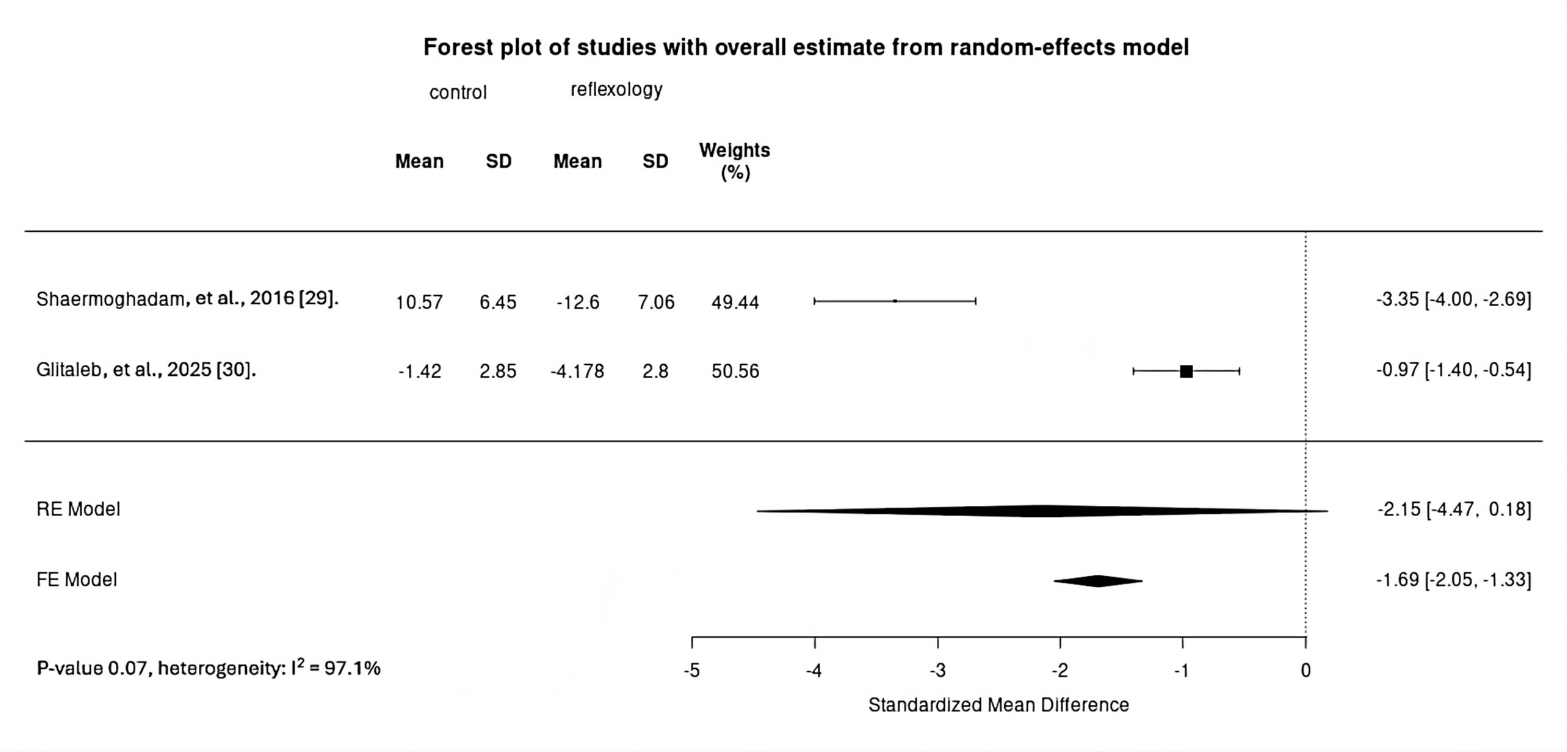
Forest plot comparing preprocedural anxiety change between reflexology and control FE, fixed-effects; RE, random-effects; SD, standard deviation

Change in anxiety level was examined in three studies [28-30]. Two studies [29,30] applied reflexology prior to endoscopy, whereas one study [28] applied abdominal massage post-endoscopy. Due to high clinical heterogeneity regarding intervention timing, the meta-analysis was limited to the reflexology studies (192 participants). Regarding measurement scales, one study [29] utilized the DASS-21, whereas the other two studies [28,30] used the STAI. The evidence was very uncertain regarding the effect of musculoskeletal manipulation (including reflexology) on pre-procedural anxiety compared with routine care: effect size (SMD) -2.15, 95% confidence intervals (CI) -4.47 to 0.18; I^2^ = 97.1%; very low certainty evidence (Figure 2). The findings from the abdominal massage study [28] (82 participants) were also very uncertain. The post-intervention STAI median values obtained from the author were 39.95 for the abdominal massage group and 40.19 for the control group. The post-intervention STAI median difference was -0.24; however, the 95% CI was not reported.

Change in abdominal pain was examined in two studies [27,28] (142 participants). One study [28] utilized the VAS, while the other [27] used the NRS. Meta-analysis found very uncertain evidence that musculoskeletal manipulation (post-colonoscopy abdominal massage) reduces abdominal pain compared with routine care. The effect size (SMD) -1.02 (95% CI -2.00 to -0.05); I^2^ = 86.7%; low certainty evidence (Figure 4).

**Figure 3:**
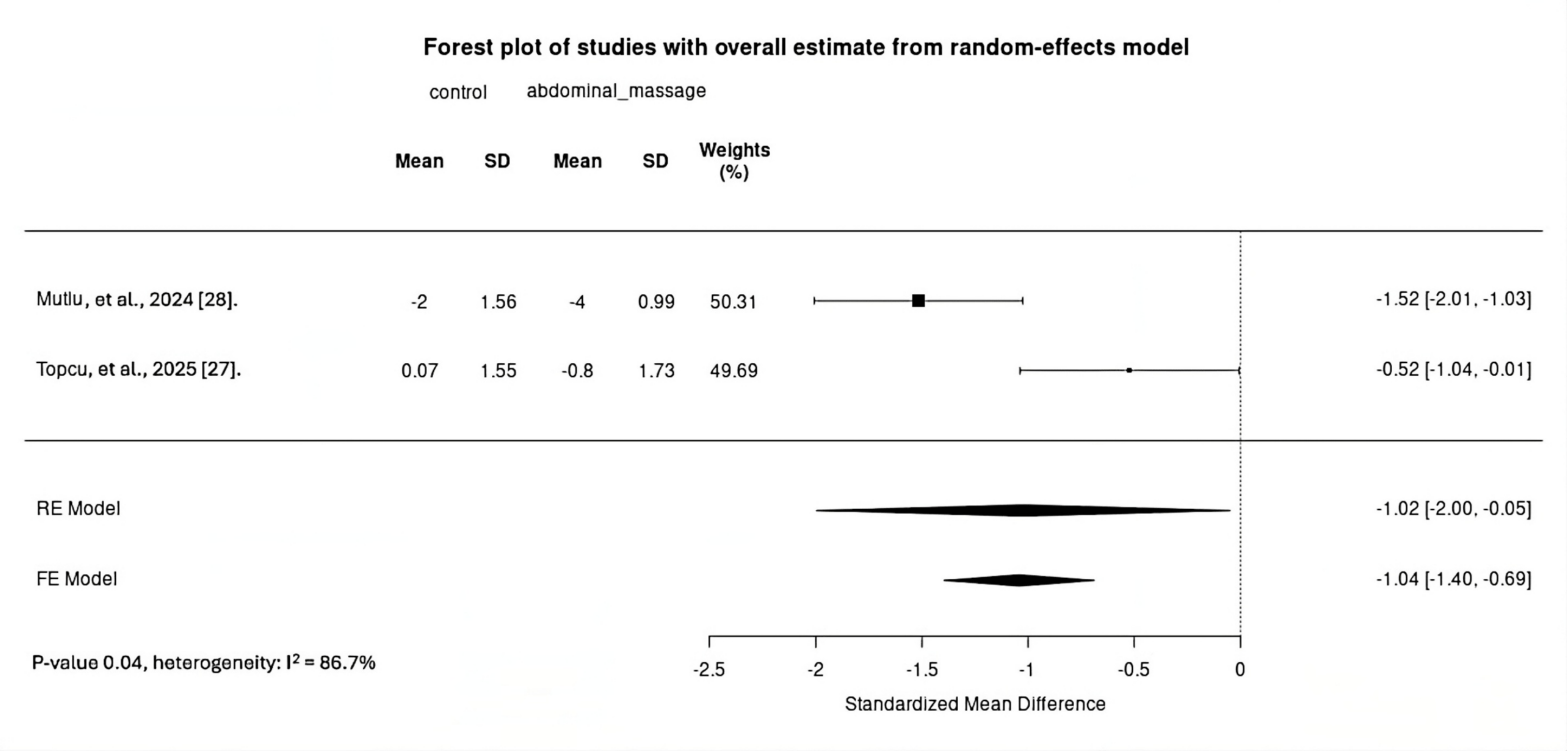
Forest plot comparing post-colonoscopy abdominal pain change between abdominal massage and control FE, fixed-effects; RE, random-effects; SD, standard deviation

Change in abdominal bloating was examined in one study [27] (60 participants). The evidence for the effect of post-colonoscopy abdominal massage on abdominal bloating compared with routine care is very uncertain. The NRS mean difference was -0.3; however, the 95% CI was not reported.

Secondary outcomes

Adverse effects were examined in two studies [27,28] (142 participants). Data on the absence of adverse events in studies [27,28] were obtained through direct inquiry to the authors. The evidence is uncertain about the adverse effects of musculoskeletal manipulation (post-colonoscopy abdominal massage) compared with routine care: 0/71 vs. 0/71 (low certainty evidence). The reflexology studies [29,30] lacked data regarding adverse effects.

Change in abdominal distension was examined in one study [28] (82 participants). The evidence is very uncertain regarding the effect of post-colonoscopy abdominal massage on abdominal distension compared with routine care. The median difference in abdominal circumference was -1.0 cm; however, the 95% CI was not reported.

Discussion

Our review revealed that the evidence for the effects of musculoskeletal manipulation (abdominal massage and reflexology) on anxiety, abdominal pain, and abdominal bloating has either low or very low certainty due to imprecision and high risk of bias. However, abdominal massage showed some potential for reducing abdominal pain [27,28]. To our knowledge, our review is the first systematic review focusing on the efficacy of musculoskeletal manipulation in patients undergoing UGIE and colonoscopy. This analysis is meaningful because it not only highlights the potential of post-colonoscopy massage but also quantitatively demonstrates the high level of uncertainty and the significant evidence gap in this field.

Given this low certainty of evidence, musculoskeletal manipulation, specifically abdominal massage and reflexology, cannot yet be definitively recommended in clinical practice for patients undergoing UGIE or colonoscopy. While the certainty of the effect is low, abdominal massage may reduce abdominal pain. One study [28] suggests that abdominal massage may lead to faster pain reduction and shorter time to baseline recovery compared to the control group. The primary discrepancy in outcomes appears to be linked to the baseline severity of abdominal pain: the effect may be stronger in participants with severe abdominal pain [28] but less effective in those with mild pain [27]. This suggests that abdominal massage may be a more clinically meaningful intervention for patients with substantial post-colonoscopy abdominal pain. Consequently, baseline symptom severity emerges as a crucial factor influencing both clinical relevance and inter-study heterogeneity.

Furthermore, heterogeneity was also influenced by the difference in practitioner type (e.g., healthcare professionals versus relatives of patients) [27,28]. The efficacy of massage performed by a professional versus a caregiver is a recognized research challenge, as the trial in another field indicates that the equivalence of their therapeutic effects is uncertain [31,32]. The potential variance in technique, experience, and patient rapport between a qualified professional and a patient’s relative contributes to a significant source of methodological uncertainty. Therefore, the observed discrepancies linked to baseline symptom severity and practitioner variability contribute to methodological uncertainty, hindering the definitive assessment of the intervention's true effect.

Beyond these practitioner- and patient-related heterogeneity factors, the fundamental methodological challenges of the included studies warrant critical assessment. First, it is unavoidable that studies in our review will have a high risk of bias due to the inherent blinding difficulties with the control group, owing to the lack of musculoskeletal manipulation. Notably, no studies were excluded from this review due to problems with the control group (e.g., sham intervention, touch massage). Given that true blinding is impossible, the design of appropriate control groups remains a fundamental methodological challenge. For a different population (back pain patients), a sham touch massage has been used in studies on massage [33], and a similar approach could be considered in this context. However, the challenge remains in designing a sham intervention that successfully blinds both the patient and the practitioner while controlling for the potential placebo effect of simple touch. Second, statistical limitations were also evident; the small sample sizes and inconsistent reporting fundamentally contribute to the inconclusive nature of the evidence. Ultimately, these combined methodological and statistical flaws demonstrate the immaturity of the current evidence base.

This review highlights the scarcity of research and the methodological limitations in studies on musculoskeletal manipulation for patients undergoing UGIE and colonoscopy. In some countries, established interventions such as sedation [9] or CO2 insufflation [34] are used to alleviate patient discomfort, which may explain the observed scarcity of research. We noted two trials explicitly mentioned the use of sedation [30] or conscious sedation [27], and the others did not report the use of either sedation or CO2 insufflation [28,29]. The lack of standardized reporting on co-interventions further contributes to methodological uncertainty. Ultimately, this reporting deficit, compounded by the potential influence of established interventions, severely impedes our ability to define the optimal participant profile for these treatments.

In addition to the aforementioned methodological constraints, heterogeneity in outcome measures presents a significant challenge to synthesizing the data. Regarding anxiety, the difference in measurement scales (DASS-21 vs. STAI) might have contributed to heterogeneity [29,30], despite similar reported anxiety levels regardless of the examination type [35]. The STAI may be suitable for assessing pre-procedural anxiety, given its use in cardiovascular interventions [15]. Based on the included studies [28-30], anxiety appears to increase before the examination and decrease afterward, suggesting that the change in pre-examination anxiety is a crucial evaluation point. Regarding abdominal pain, the heterogeneity of measurement scales and baseline levels might be a contributing factor to inconsistent findings. The frequency and severity of abdominal pain after colonoscopy have been inconsistent in past studies [36]. Patients find it difficult to express pain using a pain scale [37]; therefore, it might be difficult to assess the exact abdominal pain level. In addition, NRS and VAS are known to be correlated [38]; however, some studies have shown a discrepancy [37]. VAS may be better at capturing change than NRS when baseline values are low, while NRS is more preferred than VAS [37]. Beyond the choice of scale, defining the outcome itself is also critical. As described in the Rome IV criteria, abdominal distension (objective measurement) and bloating (subjective sensation) are different [39], and both must be considered. This significant variability in measurement tools mandates a collective effort toward standardization before meaningful data synthesis can be achieved.

Following the analysis of heterogeneity factors, the statistical and technical limitations of this review must be addressed. First, we relied on a small number of RCT studies with a high risk of bias, and our synthesis excluded other types of studies. The reliance on only two studies for each primary outcome is a major limitation. Although a meta-analysis was performed, the small number of studies and the observed high heterogeneity severely limited its statistical power and robustness. This necessitated a cautious interpretation and a strong emphasis on the qualitative discussion of the findings, consistent with the caution advised in the PRISMA guidelines [16]. The limited number of studies also prevented us from performing subgroup and sensitivity analyses to investigate the considerable statistical heterogeneity observed. Similarly, we did not assess publication bias using a funnel plot or Egger’s test due to the limited number of included studies. Second, specific technical challenges in data synthesis introduced uncertainty. Our decision to analyze the SMD of the change scores was necessary to capture the intervention's true effect on individual patient improvement, rather than simply comparing outcomes, which may be influenced by heterogeneous baseline severities [26]. However, this choice meant we required the standard deviation of the change (SD_change_). As this was often unreported in the included trials, we relied on imputing the correlation coefficient between baseline and follow-up values to estimate SD_change_ [26]. This method is consistent with the guidelines provided in the Cochrane Handbook for Systematic Reviews of Interventions [26]. This necessary imputation introduced an additional layer of statistical uncertainty and further contributes to the low certainty of the evidence reported. Third, the findings have limited generalizability, as the reflexology studies were conducted only in Iran and the abdominal massage studies only in Turkey. Furthermore, long-term follow-up data were lacking. Other limitations include unpublished protocols for comparative RCTs involving massage (we attempted to contact the authors of these protocols, but received no response), the definition of musculoskeletal manipulation, and the search strategy.

Based on the comprehensive limitations and inherent methodological challenges identified in this systematic review, we provide specific recommendations to guide the next generation of research in this field. Future research should prioritize large-scale RCTs that are adequately powered to detect statistically and clinically meaningful effects, moving beyond the inconclusive nature of the current evidence. Methodologically, trials should implement rigorous designs by considering sham interventions and ensuring the intervention is administered by qualified practitioners to minimize bias. The stratification of participants based on baseline symptom severity is essential to establish definitive conclusions and identify optimal target groups. Furthermore, to address the challenge of heterogeneity, researchers are strongly encouraged to use standardized measurement scales and ensure consistency in assessment timing. Specifically, the STAI is recommended for anxiety, while both abdominal distension and bloating should be measured for gastrointestinal symptoms. Finally, future studies should rigorously report the use of confounding co-interventions such as sedation or CO2 insufflation to enhance the clarity and generalizability of the findings, paving the way for robust subsequent meta-analyses.

## Conclusions

This review provided preliminary insights, demonstrating that evidence for musculoskeletal manipulation for patients undergoing UGIE and colonoscopy is very limited and inconclusive. While post-colonoscopy abdominal massage may reduce abdominal pain, the certainty of the evidence is low; conversely, the evidence for its effect on anxiety and abdominal bloating, as well as the effect of reflexology on pre-procedural anxiety, is very low. Therefore, there is currently no basis to recommend these interventions in clinical practice. To advance the evidence base, future research should prioritize: high-quality, large-scale studies that report effect size with 95% CI; standardizing the definition, measurement scales, and timing of outcome assessments to reduce heterogeneity; and methodologically considering both sham interventions and the stratification of participants based on baseline symptom severity to establish definitive conclusions and identify optimal target groups.

## Appendices

Appendix A

**Table 4:** PRISMA 2020 checklist for this systematic review Source: [16] PRISMA, Preferred Reporting Items for Systematic Reviews and Meta-Analyses; N/A, not applicable

Section and Topic	Item #	Checklist item		Location where item is reported
TITLE				
Title	1	Identify the report as a systematic review.		1
ABSTRACT				
Abstract	2	See the PRISMA 2020 for Abstracts checklist.		1, Appendix A
INTRODUCTION				
Rationale	3	Describe the rationale for the review in the context of existing knowledge.		1, 2
Objectives	4	Provide an explicit statement of the objective(s) or question(s) the review addresses.		1, 2
METHODS				
Eligibility criteria	5	Specify the inclusion and exclusion criteria for the review and how studies were grouped for the syntheses.		2
Information sources	6	Specify all databases, registers, websites, organisations, reference lists and other sources searched or consulted to identify studies. Specify the date when each source was last searched or consulted.		3
Search strategy	7	Present the full search strategies for all databases, registers and websites, including any filters and limits used.		Appendix B
Selection process	8	Specify the methods used to decide whether a study met the inclusion criteria of the review, including how many reviewers screened each record and each report retrieved, whether they worked independently, and if applicable, details of automation tools used in the process.		3
Data collection process	9	Specify the methods used to collect data from reports, including how many reviewers collected data from each report, whether they worked independently, any processes for obtaining or confirming data from study investigators, and if applicable, details of automation tools used in the process.		3
Data items	10a	List and define all outcomes for which data were sought. Specify whether all results that were compatible with each outcome domain in each study were sought (e.g. for all measures, time points, analyses), and if not, the methods used to decide which results to collect.		2, 3
	10b	List and define all other variables for which data were sought (e.g. participant and intervention characteristics, funding sources). Describe any assumptions made about any missing or unclear information.		3
Study risk of bias assessment	11	Specify the methods used to assess risk of bias in the included studies, including details of the tool(s) used, how many reviewers assessed each study and whether they worked independently, and if applicable, details of automation tools used in the process.		3
Effect measures	12	Specify for each outcome the effect measure(s) (e.g. risk ratio, mean difference) used in the synthesis or presentation of results.		3
Synthesis methods	13a	Describe the processes used to decide which studies were eligible for each synthesis (e.g., tabulating the study intervention characteristics and comparing against the planned groups for each synthesis (item #5)).		3, 7
	13b	Describe any methods required to prepare the data for presentation or synthesis, such as handling of missing summary statistics, or data conversions.		3
	13c	Describe any methods used to tabulate or visually display results of individual studies and syntheses.		3
	13d	Describe any methods used to synthesize results and provide a rationale for the choice(s). If meta-analysis was performed, describe the model(s), method(s) to identify the presence and extent of statistical heterogeneity, and software package(s) used.		3
	13e	Describe any methods used to explore possible causes of heterogeneity among study results (e.g. subgroup analysis, meta-regression).		3
	13f	Describe any sensitivity analyses conducted to assess robustness of the synthesized results.		3
Reporting bias assessment	14	Describe any methods used to assess risk of bias due to missing results in a synthesis (arising from reporting biases).		3
Certainty assessment	15	Describe any methods used to assess certainty (or confidence) in the body of evidence for an outcome.		2, Table 2
RESULTS				
Study selection	16a	Describe the results of the search and selection process, from the number of records identified in the search to the number of studies included in the review, ideally using a flow diagram.		4, 5, Figure 1
	16b	Cite studies that might appear to meet the inclusion criteria, but which were excluded, and explain why they were excluded.		Appendix C
Study characteristics	17	Cite each included study and present its characteristics.		4, 5, Table 1
Risk of bias in studies	18	Present assessments of risk of bias for each included study.		Table 3, Appendix D
Results of individual studies	19	For all outcomes, present, for each study: (a) summary statistics for each group (where appropriate) and (b) an effect estimate and its precision (e.g., confidence/credible interval), ideally using structured tables or plots.		Table 2, Figure 2, Figure 3
Results of syntheses	20a	For each synthesis, briefly summarise the characteristics and risk of bias among contributing studies.		7, 8
	20b	Present results of all statistical syntheses conducted. If meta-analysis was done, present for each the summary estimate and its precision (e.g., confidence/credible interval) and measures of statistical heterogeneity. If comparing groups, describe the direction of the effect.		7, 8, Table 2, Figure 2, Figure 3
	20c	Present results of all investigations of possible causes of heterogeneity among study results.		3, 10, 11
	20d	Present results of all sensitivity analyses conducted to assess the robustness of the synthesized results.		N/A
Reporting biases	21	Present assessments of risk of bias due to missing results (arising from reporting biases) for each synthesis assessed.		N/A
Certainty of evidence	22	Present assessments of certainty (or confidence) in the body of evidence for each outcome assessed.		Table 2
DISCUSSION				
Discussion	23a	Provide a general interpretation of the results in the context of other evidence.		10, 11
	23b	Discuss any limitations of the evidence included in the review.		10, 11
	23c	Discuss any limitations of the review processes used.		10, 11
	23d	Discuss implications of the results for practice, policy, and future research.		10, 11, 12
OTHER INFORMATION				
Registration and protocol	24a	Provide registration information for the review, including register name and registration number, or state that the review was not registered.		2
	24b	Indicate where the review protocol can be accessed, or state that a protocol was not prepared.		2
	24c	Describe and explain any amendments to information provided at registration or in the protocol.		N/A
Support	25	Describe sources of financial or non-financial support for the review, and the role of the funders or sponsors in the review.		20
Competing interests	26	Declare any competing interests of review authors.		20
Availability of data, code and other materials	27	Report which of the following are publicly available and where they can be found: template data collection forms; data extracted from included studies; data used for all analyses; analytic code; any other materials used in the review.		N/A
Section and Topic		Item #	Checklist item	Reported (Yes/No)
TITLE				
Title		1	Identify the report as a systematic review.	Yes
BACKGROUND				
Objectives		2	Provide an explicit statement of the main objective(s) or question(s) the review addresses.	Yes
METHODS				
Eligibility criteria		3	Specify the inclusion and exclusion criteria for the review.	Yes
Information sources		4	Specify the information sources (e.g., databases, registers) used to identify studies and the date when each was last searched.	Yes
Risk of bias		5	Specify the methods used to assess risk of bias in the included studies.	No
Synthesis of results		6	Specify the methods used to present and synthesise results.	No
RESULTS				
Included studies		7	Give the total number of included studies and participants and summarise relevant characteristics of studies.	No
Synthesis of results		8	Present results for main outcomes, preferably indicating the number of included studies and participants for each. If meta-analysis was done, report the summary estimate and confidence/credible interval. If comparing groups, indicate the direction of the effect (i.e., which group is favoured).	No
DISCUSSION				
Limitations of evidence		9	Provide a brief summary of the limitations of the evidence included in the review (e.g., study risk of bias, inconsistency and imprecision).	Yes
Interpretation		10	Provide a general interpretation of the results and important implications.	Yes
OTHER				
Funding		11	Specify the primary source of funding for the review.	No
Registration		12	Provide the register name and registration number.	Yes

Appendix B

**Table 5:** Detailed search strategy CENTRAL, Cochrane Central Register of Controlled Trials; CINAHL, Cumulative Index to Nursing and Allied Health Literature; EMBASE, Excerpta Medica database; ICTRP, International Clinical Trials Registry Platform; PEDro, Physiotherapy Evidence Database

Search formula for MEDLINE (PubMed) conducted on July 16, 2025
#1 Colonoscopy[mh]
#2 Sigmoidoscopy[mh]
#3 Duodenoscopy[mh]
#4 Esophagoscopy[mh]
#5 Gastroscopy[mh]
#6 Colonoscopy[tiab]
#7 Sigmoidoscopy[tiab]
#8 Duodenoscopy[tiab]
#9 Esophagoscopy[tiab]
#10 Gastroscopy[tiab]
#11 “upper endoscopy”[tiab]
#12 “upper gastrointestinal endoscopy”[tiab]
#13 “upper GI endoscopy”[tiab]
#14 esophagogastroduodenoscopy[tiab]
#15 colonofiberscopy[tiab]
#16 “Endoscopy, Gastrointestinal”[mh]
#17 #1 OR #2 OR #3 OR #4 OR #5 OR #6 OR #7 OR #8 OR #9 OR #10 OR #11 OR #12 OR #13 OR #14 OR #15 OR #16
#18 Musculoskeletal Manipulations[mh]
#19 “Musculoskeletal Manipulation*”[tiab]
#20 Reflexology[tiab]
#21 “Manipulation Therap*”[tiab]
#22 “Manipulative Therap*”[tiab]
#23 “Manual Therap*”[tiab]
#24 Bodywork*[tiab]
#25 Rolfing[tiab]
#26 Chiropractic[tiab]
#27 Osteopathic[tiab]
#28 Acupressure[tiab]
#29 Shiatsu[tiab]
#30 “Zhi Ya”[tiab]
#31 “Zone Therap*”[tiab]
#32 Massage[tiab]
#33 #18 OR #19 OR #20 OR #21 OR #22 OR #23 OR #24 OR #25 OR #26 OR #27 OR #28 OR #29 OR #30 OR #31 OR #32
#34 #17 AND #33
Search formula for CENTRAL (Cochrane Library) conducted on July 16, 2025
[mh Colonoscopy]
[mh Sigmoidoscopy]
[mh Duodenoscopy]
[mh Esophagoscopy]
[mh Gastroscopy]
Colonoscopy:ti,ab
Sigmoidoscopy:ti,ab
Duodenoscopy:ti,ab
Esophagoscopy:ti,ab
Gastroscopy:ti,ab
"upper endoscopy":ti,ab
"upper gastrointestinal endoscopy":ti,ab
"upper GI endoscopy":ti,ab
esophagogastroduodenoscopy:ti,ab
colonofiberscopy:ti,ab
#1 OR #2 OR #3 OR #4 OR #5 OR #6 OR #7 OR #8 OR #9 OR #10 OR #11 OR #12 OR #13 OR #14 OR #15 OR #16
[mh "Musculoskeletal Manipulations"]
("Musculoskeletal" NEXT Manipulation*):ti,ab
[mh Reflexology]
Reflexology:ti,ab
("Manipulation" NEXT Therap*):ti,ab
("Manipulative" NEXT Therap*):ti,ab
("Manual" NEXT Therap*):ti,ab
Bodywork*:ti,ab
Rolfing:ti,ab
Chiropractic:ti,ab
Osteopathic:ti,ab
Acupressure:ti,ab
Shiatsu:ti,ab
"Zhi Ya":ti,ab
("Zone" NEXT Therap*):ti,ab
Massage:ti,ab
#18 OR #19 OR #20 OR #21 OR #22 OR #23 OR #24 OR #25 OR #26 OR #27 OR #28 OR #29 OR #30 OR #31 OR #32 OR #33
#17 AND #34
Search formula for EMBASE (Dialog) conducted on July 16, 2025
S1: (EMB.EXACT.EXPLODE("Colonoscopy") OR EMB.EXACT.EXPLODE("Sigmoidoscopy") OR EMB.EXACT.EXPLODE("Duodenoscopy") OR EMB.EXACT.EXPLODE("Esophagoscopy") OR EMB.EXACT.EXPLODE("Gastroscopy") OR TI(Colonoscopy) OR AB(Colonoscopy) OR TI(Sigmoidoscopy) OR AB(Sigmoidoscopy) OR TI(Duodenoscopy) OR AB(Duodenoscopy) OR TI(Esophagoscopy) OR AB(Esophagoscopy) OR TI(Gastroscopy) OR AB(Gastroscopy) OR TI("upper endoscopy") OR AB("upper endoscopy") OR TI("upper gastrointestinal endoscopy") OR AB("upper gastrointestinal endoscopy") OR TI("upper GI endoscopy") OR AB("upper GI endoscopy") OR TI(esophagogastroduodenoscopy) OR AB(esophagogastroduodenoscopy) OR TI(colonofiberscopy) OR AB(colonofiberscopy) OR EMB.EXACT.EXPLODE("Endoscopy, Gastrointestinal"))
S2: (EMB.EXACT.EXPLODE("Musculoskeletal Manipulations") OR TI("Musculoskeletal Manipulation*") OR AB("Musculoskeletal Manipulation*") OR TI(Reflexology) OR AB(Reflexology) OR TI("Manipulation Therap*") OR AB("Manipulation Therap*") OR TI("Manipulative Therap*") OR AB("Manipulative Therap*") OR TI("Manual Therap*") OR AB("Manual Therap*") OR TI(Bodywork*) OR AB(Bodywork*) OR TI(Rolfing) OR AB(Rolfing) OR TI(Chiropractic) OR AB(Chiropractic) OR TI(Osteopathic) OR AB(Osteopathic) OR TI(Acupressure) OR AB(Acupressure) OR TI(Shiatsu) OR AB(Shiatsu) OR TI("Zhi Ya") OR AB("Zhi Ya") OR TI("Zone Therap*") OR AB("Zone Therap*") OR TI(Massage) OR AB(Massage))
S3: S1 AND S2
Search formula for ICTRP conducted on July 17, 2025
(Colonoscopy OR CS OR Colonofiberscopy OR CF OR Sigmoidoscopy OR FS OR SF OR Duodenoscopy OR Esophagoscopy OR Gastroscopy OR “Upper endoscopy” OR “Upper gastrointestinal endoscopy” OR “Upper GI endoscopy” OR UGIE OR Esophagogastroduodenoscopy OR EGD)
AND
(“Musculoskeletal Manipulation” OR Reflexology OR “Manipulation Therapy” OR “Manipulation Therapies” OR “Manipulative Therapy” OR “Manipulative Therapies” OR “Manual Therapy” OR “Manual Therapies” OR Bodywork OR Rolfing OR Chiropractic OR Osteopathic OR Acupressure OR Shiatsu OR “Zhi Ya” OR “Zone Therapy” OR “Zone therapies” OR Massage)
Search formula for ClinicalTrial.gov conducted on July 17, 2025
(Colonoscopy OR Colonofiberscopy OR Sigmoidoscopy OR Duodenoscopy OR Esophagoscopy OR Gastroscopy OR “Upper endoscopy” OR “Upper gastrointestinal endoscopy” OR “Upper GI endoscopy” OR Esophagogastroduodenoscopy)
AND
(“Musculoskeletal Manipulation” OR Reflexology OR “Manipulation Therapy” OR “Manipulation Therapies” OR “Manipulative Therapy” OR “Manipulative Therapies” OR “Manual Therapy” OR “Manual Therapies” OR Bodywork OR Rolfing OR Chiropractic OR Osteopathic OR Acupressure OR Shiatsu OR “Zhi Ya” OR “Zone Therapy” OR “Zone Therapies” OR Massage)
Search formula for CINAHL conducted on July 16, 2025
(MH Colonoscopy+)
(MH Sigmoidoscopy+)
(MH Duodenoscopy+)
(MH Esophagoscopy+)
(MH Gastroscopy+)
(TI Colonoscopy OR AB Colonoscopy)
(TI Sigmoidoscopy OR AB Sigmoidoscopy)
(TI Duodenoscopy OR AB Duodenoscopy)
(TI Esophagoscopy OR AB Esophagoscopy)
(TI Gastroscopy OR AB Gastroscopy)
(TI "upper endoscopy" OR AB "upper endoscopy")
(TI "upper gastrointestinal endoscopy" OR AB "upper gastrointestinal endoscopy")
(TI "upper GI endoscopy" OR AB "upper GI endoscopy")
(TI esophagogastroduodenoscopy OR AB esophagogastroduodenoscopy)
(TI colonofiberscopy OR AB colonofiberscopy)
(MH "Endoscopy, Gastrointestinal+")
S1 OR S2 OR S3 OR S4 OR S5 OR S6 OR S7 OR S8 OR S9 OR S10 OR S11 OR S12 OR S13 OR S14 OR S15 OR S16
(MH "Musculoskeletal Manipulations+")
(TI "Musculoskeletal Manipulation*" OR AB "Musculoskeletal Manipulation*")
(MH Reflexology+)
(TI Reflexology OR AB Reflexology)
(TI "Manipulation Therap*" OR AB "Manipulation Therap*")
(TI "Manipulative Therap*" OR AB "Manipulative Therap*")
(TI "Manual Therap*" OR AB "Manual Therap*")
(TI Bodywork* OR AB Bodywork*)
(TI Rolfing OR AB Rolfing)
(TI Chiropractic OR AB Chiropractic)
(TI Osteopathic OR AB Osteopathic)
(TI Acupressure OR AB Acupressure)
(TI Shiatsu OR AB Shiatsu)
(TI "Zhi Ya" OR AB "Zhi Ya")
(TI "Zone Therap*" OR AB "Zone Therap*")
(TI Massage OR AB Massage)
S18 OR S19 OR S20 OR S21 OR S22 OR S23 OR S24 OR S25 OR S26 OR S27 OR S28 OR S29 OR S30 OR S31 OR S32 OR S33
S17 AND S34
Search formula for PEDro conducted on July 16, 2025
In the Advanced Search, input and search for each of the following terms in the Abstract & Title fields: Colonoscopy, Colonofiberscopy, Sigmoidoscopy, Duodenoscopy, Esophagoscopy, Gastroscopy, Upper endoscopy, Upper gastrointestinal endoscopy, Upper GI endoscopy, Esophagogastroduodenoscopy.

Appendix C

**Table 6:** List of excluded studies and reasons for exclusion ICTRP, International Clinical Trials Registry Platform

Reason for exclusion	Title
Wrong design	Jo WH, Kim MS: The effects of abdominal meridian massage on abdominal distention, pain, and recovery of bowel motility after sedated colonoscopy. [Article in Korean]. J Korean Acad Fundam Nurs. 2022:12-23. 10.7739/jkafn.2022.29.1.12
Wrong population	Zhao X, Xiao Q, Li T, Chen H: Application of Ziwuliuzhu timed acupoint massage in bowel preparation before colonoscopy. [Article in Chinese]. Nurs Integr Tradit Chin & West Med. 2019:59. http://218.28.6.71:81/Qikan/Article/Detail?id=7100636956
Wrong population	ICTRP: Auricular acupressure to reduce the incidence of adverse reactions to colonoscopic bowel preparation: a randomized controlled trial protocol. (2022). Accessed: July 27, 2025: https://trialsearch.who.int/Trial2.aspx?TrialID=ChiCTR2200061742
Wrong population	Zhang J, Liu C, Ruan G, Zhang H, Zhang B, Hu X, Zhong C: Auricular acupressure for minimizing adverse reactions to colonoscopic bowel preparation in hospitalized patients: A randomized controlled trial. Heliyon. 2025, 1100567:5. 10.1016/j.heliyon.2025.e42187
Wrong intervention	Tarçin O, Gürbüz AK, Pocan S, Keskin Ö, Demirtürk L. Acustimulation of the Neiguan point during gastroscopy: its effects on nausea and retching. Turk J Gastroenterol. 2004:258-262. https://tjg.avesjournals.com/public/pdfs/buyuk/3631.pdf
Protocols without results	ClinicalTrials.gov: Foot and abdominal massage applied to after colonoscopy. (2024). Accessed: July 27, 2025: https://clinicaltrials.gov/study/NCT06333535?term=NCT06333535&rank=1
Protocols without results	ClinicalTrials.gov: The effect of post colonoscopy abdominal massage on abdominal pain, distension, discomfort and patient satisfaction. (2020). Accessed: July 27, 2025: https://clinicaltrials.gov/study/NCT04979351?term=NCT04979351&rank=1
Protocols without results	ICTRP: Study on the effect of refined nursing on abdominal discomfort in patients undergoing painless colonoscopy. (2024). Accessed: July 27, 2025: https://trialsearch.who.int/Trial2.aspx?TrialID=ChiCTR2400094239.
Protocols without results	ICTRP: Study on the effect of refined nursing on abdominal discomfort in patients undergoing painless colonoscopy. (2024). Accessed: July 27, 2025: https://trialsearch.who.int/Trial2.aspx?TrialID=ChiCTR2300070031.
An abstract awaiting classification (as the report was not obtainable in Japan)	Liang QH, Qu S, Zhao LR, Li Y, Xie YX. Observation on relieving side effects in gastroscopy by point massage. [Article in Chinese]. Chin J Nurs. 1994:298-299.

Appendix D

**Table 7:** Detailed Risk of Bias 2.0 (RoB 2.0) assessment Domains of RoB 2.0: D1: Bias arising from the randomization process. D2: Bias due to deviations from intended intervention. D3: Bias due to missing outcome data. D4: Bias in measurement of the outcome. D5: Bias in selection of the reported result. We directed two separate inquiries to the author [29] regarding patient demographics, randomization methods, blinding methods, and allocation concealment. Unfortunately, these attempts remained unanswered. ITT, intention to treat; RoB 2.0, Risk of Bias 2.0

Study	Domain and reason for some concerns/high risk
Shaermoghadam et al., 2016 [29]	D2: Randomization/allocation concealment not described. D3: 5/95 participants undescribed. D4: Outcome assessors not blinded. D5: Protocol planned 3 time points, only 2 reported.
Golitaleb et al., 2025 [30]	D2: Dropouts suggested incomplete ITT analysis. D4: Outcome assessors not blinded.
Mutlu et al., 2024 [28]	D1: Differences in reasons for colonoscopy among groups. D4: Outcome assessors not blinded. D5: Protocol was not registered.
Topcu et al., 2025 [27]	D4: Outcome assessors not blinded.
